# Identification of compelling inhibitors of human norovirus 3CL protease to combat gastroenteritis: A structure-based virtual screening and molecular dynamics study

**DOI:** 10.3389/fchem.2022.1034911

**Published:** 2022-09-30

**Authors:** Shan He, Alaa F. Nahhas, Alaa Hamed Habib, Mohammed Ali Alshehri, Saleh Alshamrani, Saeed A. Asiri, Mashael M. Alnamshan, Nawal Helmi, Ibtesam Al-Dhuayan, Jawaher Almulhim, Ahmed M. Alharbi, Dongxiao Su, Ankita Kumari, Abdul Rahaman

**Affiliations:** ^1^ School of Chemistry and Chemical Engineering, Guangzhou University, Guangzhou, China; ^2^ Institute for Nano Scale and Technology, College of Science and Engineering, Flinders University, Adelaide, SA, Australia; ^3^ Suzhou Ultra-Water-Cleaning Tech, Pty, Ltd., Suzhou, Jiangsu, China; ^4^ Biochemistry Department, Faculty of Science, King Abdulaziz University, Jeddah, Saudi Arabia; ^5^ Department of Physiology, Faculty of Medicine, King Abdulaziz University, Jeddah, Saudi Arabia; ^6^ Department of Clinical Laboratory Sciences, Faculty of Applied Medical Sciences, Najran University, Najran, Saudi Arabia; ^7^ Department of Biology, College of Science, Imam Abdulrahman Bin Faisal University, Dammam, Saudi Arabia; ^8^ Department of Biochemistry, College of Science, University of Jeddah, Jeddah, Saudi Arabia; ^9^ Department of Medical Laboratory Technology, College of Applied Medical Sciences, University of Jeddah, Jeddah, Saudi Arabia; ^10^ Department of Biology, College of Science, Imam Abdulrahman Bin Faisal University, Dammam, Saudi Arabia; ^11^ Department of Biological Sciences, King Faisal University, Alahsa, Saudi Arabia; ^12^ Department of Clinical Laboratory Sciences, College of Applied Medical Sciences, University of Hail, Hail, Saudi Arabia; ^13^ School of Food Science and Engineering, South china University of Technology, Guangzhou, China

**Keywords:** noroviruses, gastroenteritis, protease, natural compounds, molecular dynamics

## Abstract

Human noroviruses (NV) are the most prevalent cause of sporadic and pandemic acute gastroenteritis. NV infections cause substantial morbidity and death globally, especially amongst the aged, immunocompromised individuals, and children. There are presently no authorized NV vaccines, small-molecule therapies, or prophylactics for humans. NV 3 C L protease (3CLP) has been identified as a promising therapeutic target for anti-NV drug development. Herein, we employed a structure-based virtual screening method to screen a library of 700 antiviral compounds against the active site residues of 3CLP. We report three compounds, Sorafenib, YM201636, and LDC4297, that were revealed to have a higher binding energy (BE) value with 3CLP than the control (Dipeptidyl inhibitor 7) following a sequential screening, in-depth molecular docking and visualization, physicochemical and pharmacological property analysis, and molecular dynamics (MD) study. Sorafenib, YM201636, and LDC4297 had BEs of -11.67, -10.34, and -9.78 kcal/mol with 3CLP, respectively, while control had a BE of -6.38 kcal/mol. Furthermore, MD simulations of the two best compounds and control were used to further optimize the interactions, and a 100 ns MD simulation revealed that they form stable complexes with 3CLP. The estimated physicochemical, drug-like, and ADMET properties of these hits suggest that they might be employed as 3CLP inhibitors in the management of gastroenteritis. However, wet lab tests are a prerequisite to optimize them as NV 3CLP inhibitors.

## Introduction

Human noroviruses (NV), which are members of the Caliciviridae family, are the leading cause of acute gastroenteritis globally, with substantial morbidity and a significant economic burden (Koo et al., 2010; Lopman et al., 2016). NV infections are difficult to combat because of their easy food and waterborne transmission, their genomic diversity, and environmental stability (Hall, 2012). The situation is aggravated further by the absence of diagnostics and NV-specific treatments and prophylactics, such as vaccinations (Kaufman et al., 2014; Rocha-Pereira et al., 2014). Therefore, the discovery of anti-NV small-molecule therapies and prophylactics, as well as efficient vaccinations, is an imperative and unmet medical need.

The NV genome is made up of a positive-strand RNA with three open reading frames encoding: i) polyprotein, ii) minor capsid protein, and iii) main capsid protein. The polyprotein is processed by a virally encoded 3 C L protease (3CLP), a cysteine protease having cysteine139-histidin30-glutamate54 catalytic triad, an extended binding cleft, and a major substrate selectivity for a P_1_ glutamine (or glutamate) residue, yielding six nonstructural proteins required for NV replication (Hussey et al., 2011; Muhaxhiri et al., 2013). NV 3CLP is important in the virus’s life cycle, making it ideal for the development/discovery of anti-norovirus treatments and prophylactics (Chang et al., 2019; Netzler et al., 2019). Peptidyl and macrocyclic transition state inhibitors, as well as transition state mimics, are only a few of the 3CLpro inhibitors that have been found to exhibit anti-NV activity (Deng et al., 2013; Galasiti Kankanamalage et al., 2015; Damalanka et al., 2016; Weerawarna et al., 2016). In addition, a dipeptidyl transition state inhibitor of the NV 3CLpro has also been shown to work in a mouse model (Galasiti Kankanamalage et al., 2015).

Traditional drug development is time-consuming and costly, taking an average of 10–15 years to reach the market and costing an probably 58.8 billion USD in 2015 (Mullard, 2016). These figures represent a dramatic 10% rise over previous years for both the biotechnology and pharmaceutical sectors. The high failure rate and high expense of this conventional approach to drug development have necessitated the adoption of computer-assisted drug development (Schaduangrat et al., 2020). The various adverse effects of drugs that result in severe toxicity necessitate the screening of drug likeness and physicochemical properties at the early stage of drug development process to maximize success and minimize the time spent screening candidates (Hughes et al., 2011). Here, we aimed to find novel NV 3CLP inhibitors using the *in silico* approach to combat gastroenteritis.

## Methodology

### Retrieval and preparation of 3CLP and compound library

The 3D structure of 3CLP, which has the PDB ID 5T6F, was taken from the protein data bank (PDB) (Galasiti Kankanamalage et al., 2017). In order to clean up the complex, the hetero atoms and water molecules had to be removed. Whereupon, using the steepest descent method with an RMS gradient of 0.1, energy minimization of protein was performed for 1,000 steps. This study employs a unique collection of 700 compounds known to target HCV protease, HIV protease, Integrase, Reverse Transcriptase, and other enzymes, including some FDA-approved compounds. The antiviral compounds were retrieved in ‘sdf’ format, prepared by minimization, and converted to ‘pdbqt’ format for virtual screening (VS.).

### Structure-based virtual screening

The prepared library of compounds in pdbqt format was used for VS. against the active site residues of the 3CLP with the PyRx 0.8 program (Dallakyan and Olson, 2015). The grid center of the protein was set as X = 4.963, Y = 67.188, and Z = -6.787.

### Molecular docking simulations

The top 20 compounds, including the control (Dipeptidyl inhibitor 7) (Supplementary Table S1), were subjected to an in-depth molecular docking simulation following the virtual screening to optimize the binding conformations of these compounds. Autodock4.2 was used to do a docking analysis with the default settings and the same grid center as virtual screening.

### Prediction of physicochemical, drug-likeness, and ADMET properties

By identifying lead molecules, computational approaches help improve the success rate of experimental drug trials. The computational prediction of pharmacokinetic and ADMET properties of small molecules provides the clue to narrowing down the screening and their potential to be drug-like molecules. The efficacy and safety profiles of the selected hits and their pharmacokinetics were predicted using SwissADME (Daina et al., 2017) and the DataWarrior tool (López-López et al., 2019).

The attributes of the bioactivity score predict the overall potential of the three best selected hits to be an effective lead candidate. An online tool ‘Molinspiration chemoinformatics’ (https://www.molinspiration.com), was used to evaluate the drug score of selected hits in relation to various human receptors such as ion channels, GPCRs, enzymes, kinases, proteases, and nuclear receptors. On the whole, a greater bioactivity score specifies that the active compound is more likely to be active.

### Molecular dynamics simulation

GROMACS 5.1.2 (Van Der Spoel et al., 2005) was used for MD simulations on 3CLP -control, 3 C L Protease-Sorafenib, and 3CLP -YM201636 at 300 K, with the GROMOS96 43a1 force-field (Pol-Fachin et al., 2009). The PRODRG server was used to produce the compound’s topology and force-field parameters (Schuttelkopf and Van Aalten, 2004). Charges were manually corrected in the topology file, new compound atoms were added to the complex topology files, and all of the compounds’ attributes were included in the system topology. 3CLP-control, 3CLP-Sorafenib, and 3CLP -YM201636 were immersed in a ‘cubic box’ of water molecules with an initial diameter of 8 nm using the ‘gmx editconf’ module for boundary conditions and the ‘gmx solvate’ module for solvation. Adding Na+ and Cl-ions to preserve neutrality and a physiological concentration using the gmx genion module (0.15 M) neutralized the charges on the complexes. PyMOL and VMD have been used to generate all visualizations of the 3D models (Humphrey et al., 1996; Amarnath Jonniya et al., 2021).

## Results and discussion

**FIGURE 1:**
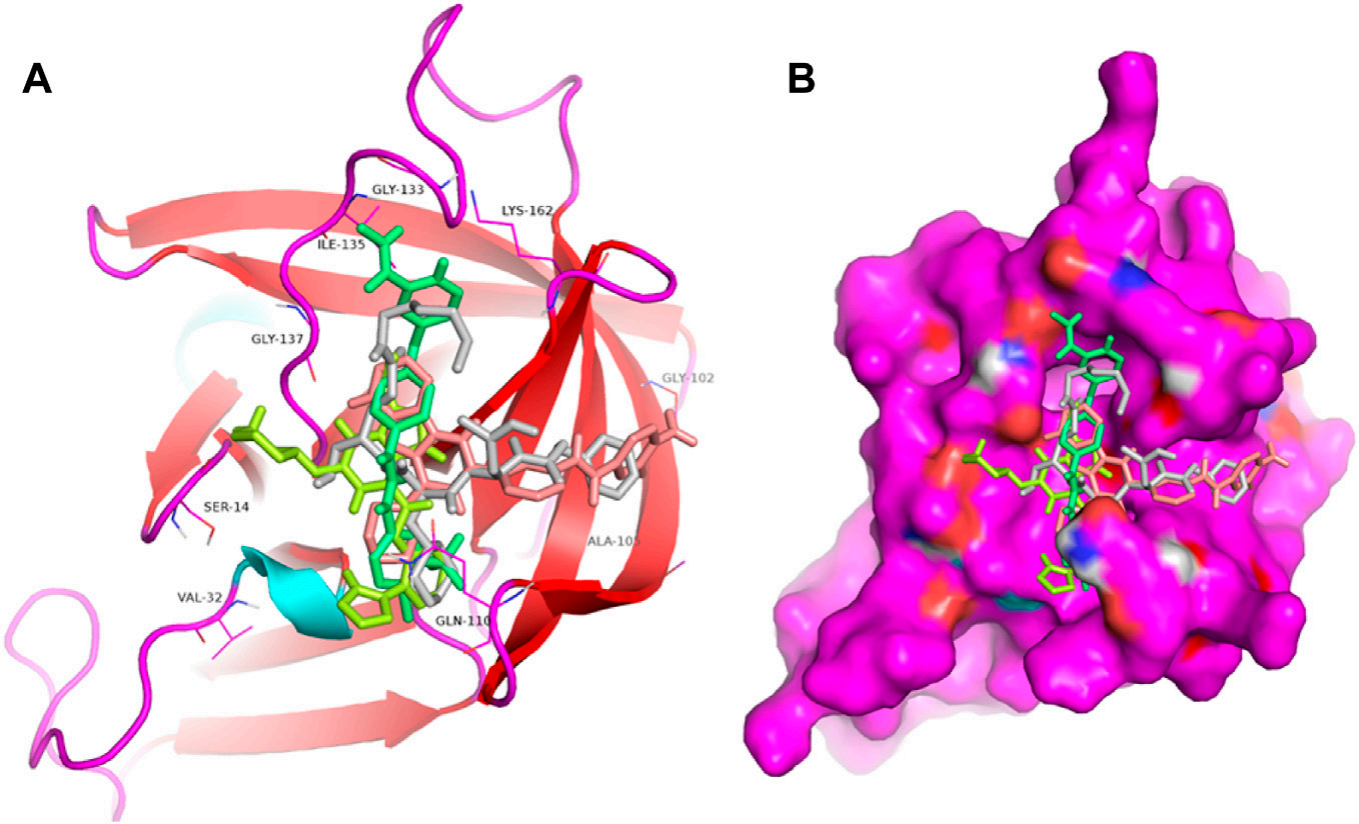
Structural alignment of screened lead compounds (sorafenib, LDC4297, YM201636) and dipeptidyl inhibitor seven in the 3CLP binding pocket **(A,B)**.

**FIGURE 2:**
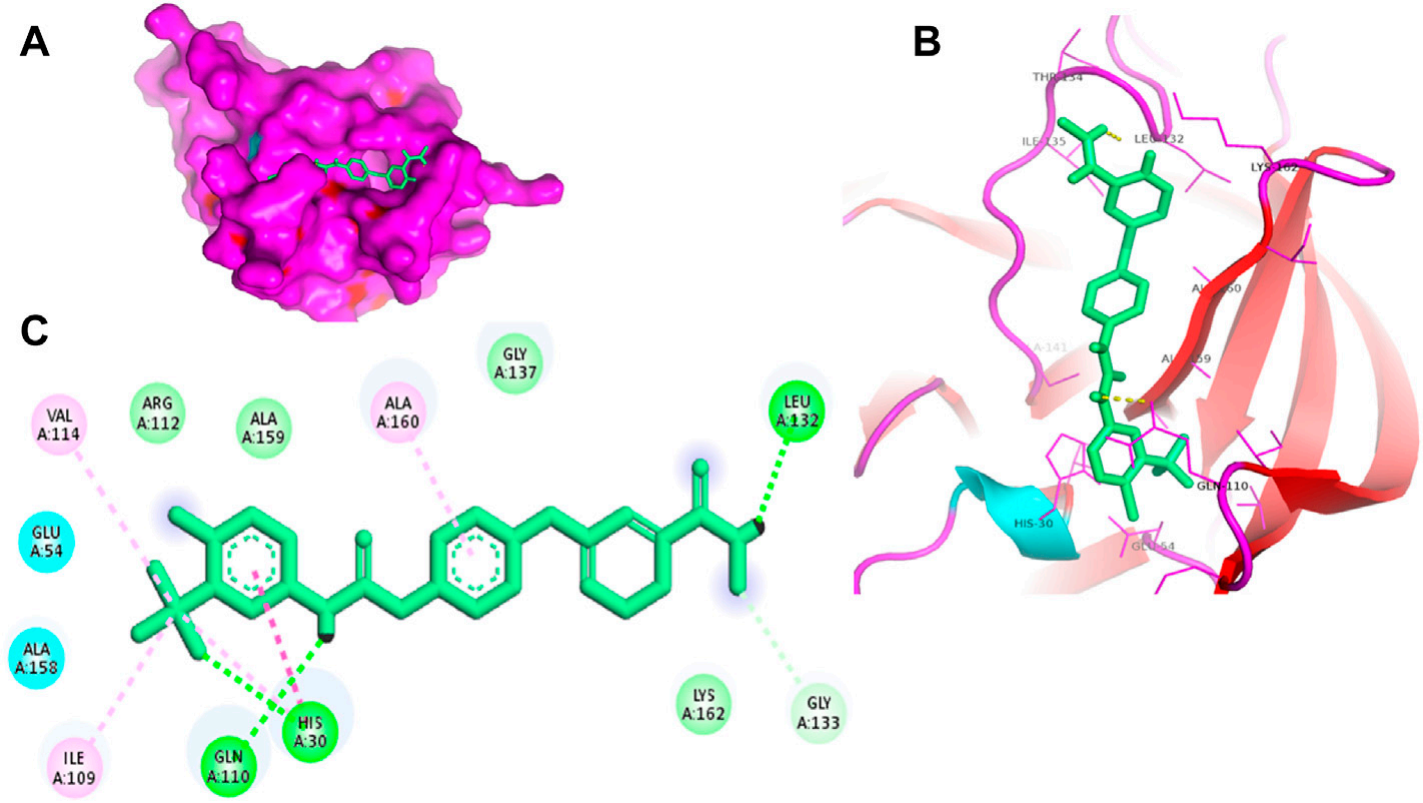
Surface view of sorafenib in the 3CLP binding pocket **(A)**, 3D **(B)** and 2D **(C)** interacting residues of 3CLP with sorafenib.

**FIGURE 3:**
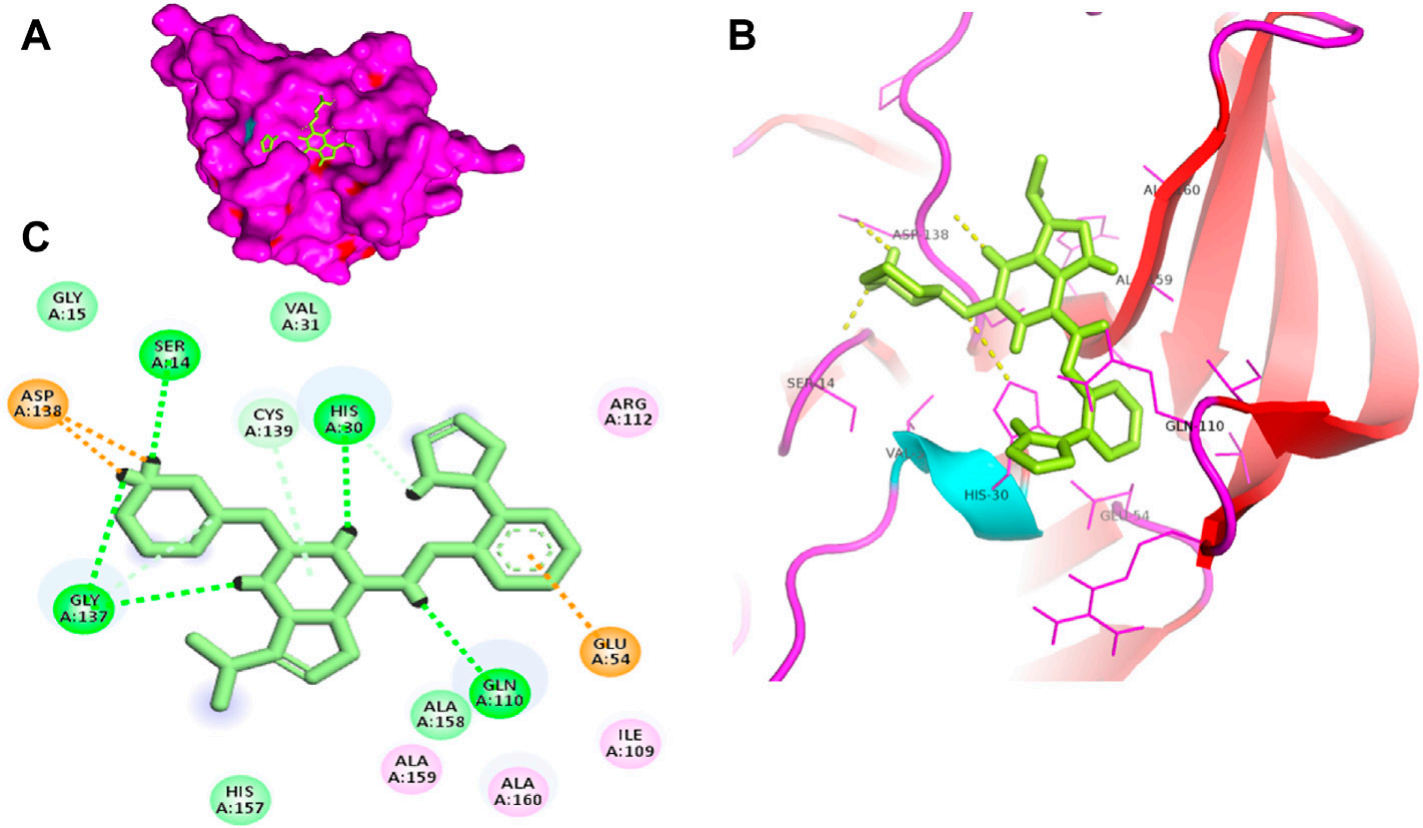
Surface view of LDC4297 in the 3CLP binding pocket **(A)**, 3D **(B)** and 2D **(C)** interacting residues of 3CLP with LDC4297.

**FIGURE 4:**
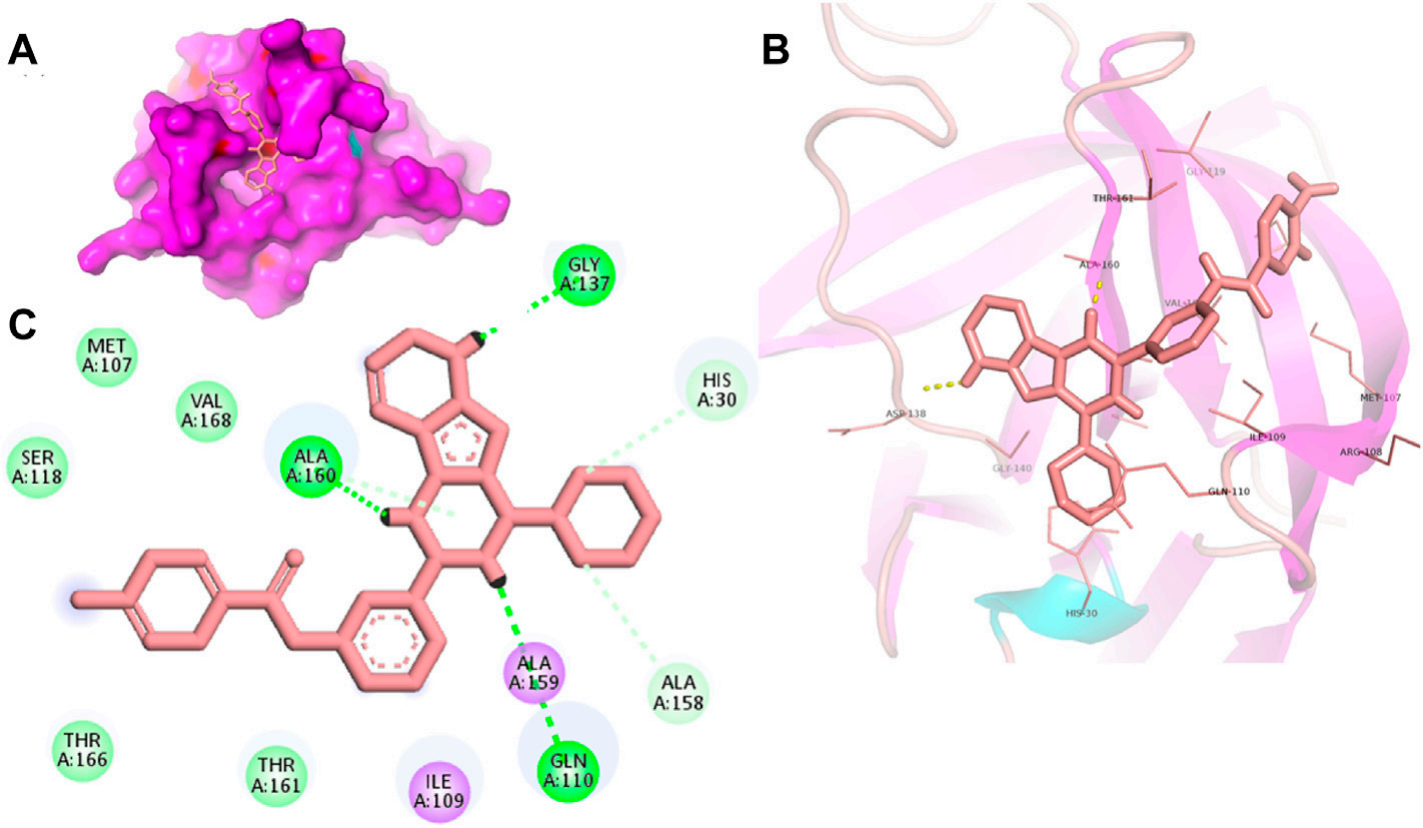
Surface view of YM201636 in the 3CLP binding pocket **(A)**, 3D **(B)** and 2D **(C)** interacting residues of 3CLP with YM201636.

Proteases are a type of enzyme that plays an important role in a several biological processes in living organisms ranging from viruses to mammals. NV 3CLP is a major viral target for anti-NV drug development due to its critical role in viral replication (Thorne and Goodfellow, 2014). This study screened 700 antiviral compounds against NV 3CLP. Sorafenib, LDC4297, and YM201636 were identified as potential lead compounds after sequential screening and interaction analysis of the complexes, as they interacted strongly with 3CLP (Figure 1). Sorafenib interacted with Glu54, Ile109, Gln110, Arg112, Val114, Leu132, Gly133, Thr134, Gly137, Ala158, His30, Ala159, Ala160, and Lys162 residues of 3CLP with a binding energy (BE) of -11.67 kcal/mol (Figure 2; Table 1). LDC4297 had a BE of -9.78 kcal/mol, and interacted with Thr28, His30, Glu54, Arg108, Ile109, Gln110, Arg112, Val114, Ile135, Pro136, Gly137, Cys139, Ala158, Ala159, Ala160, Thr161, Lys162, and Val168 residues of 3CLP (Figure 3; Table 1). Further, YM201636 interacted with His30, Ala105, Met107, Arg108, Ile109, Gln110, Ser118, Thr134, Ile135, Pro136, His157, Ala158, Ala159, Ala160, Thr161, Lys162, and Val168 residues, and has a BE of -10.34 kcal/mol with 3CLP (Figure 4; Table 1). The 3CLP protease residues Ala158, Ala160, Val168, and Ile109 have been shown to important in inhibitor binding (Galasiti Kankanamalage et al., 2017). Consistent with this, the hits Sorafenib, LDC4297, and YM201636 have been found to interact with these 3CLP residues.

**TABLE 1:** BE of lead compounds with NV 3CLP.

Compounds	Structure	Binding energy (kcal/mol)	H-bonds interacting resides
Sorafenib	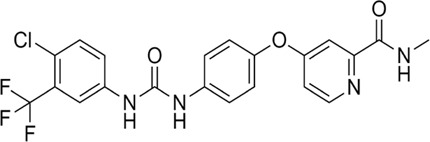	-11.67	His30, Gln110, and Leu132
LDC4297	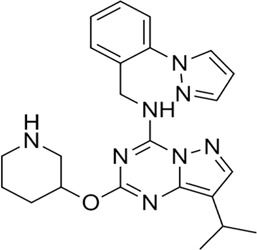	-9.78	Ala158, and Ala160
YM201636	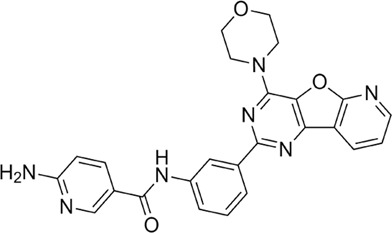	-10.34	His30 and Ala160
Dipeptidyl inhibitor 7^a^	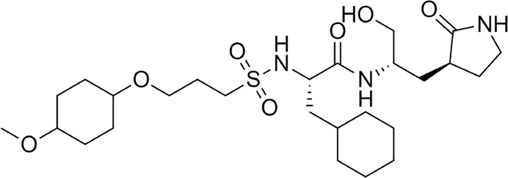	-6.38	His30, Gln110, Thr134, Ala158, and Ala160

a Reference inhibitor.

NV 3CLP’s mode of action is similar to that of comparable cysteine proteases, in which Cys139 functions as a nucleophile, His30 acts as a general acid/base, and Glu54 aids in the alignment of His30 and stimulates the deprotonation of Cys139 (Chang et al., 2019). Interestingly, this study showed that the lead compounds sorafenib, LDC4297, and YM201636 interacted with these residues (His30, Glu54, and Cys139) of NV 3CLP, possibly inhibiting the 3CLP.

**FIGURE 5:**
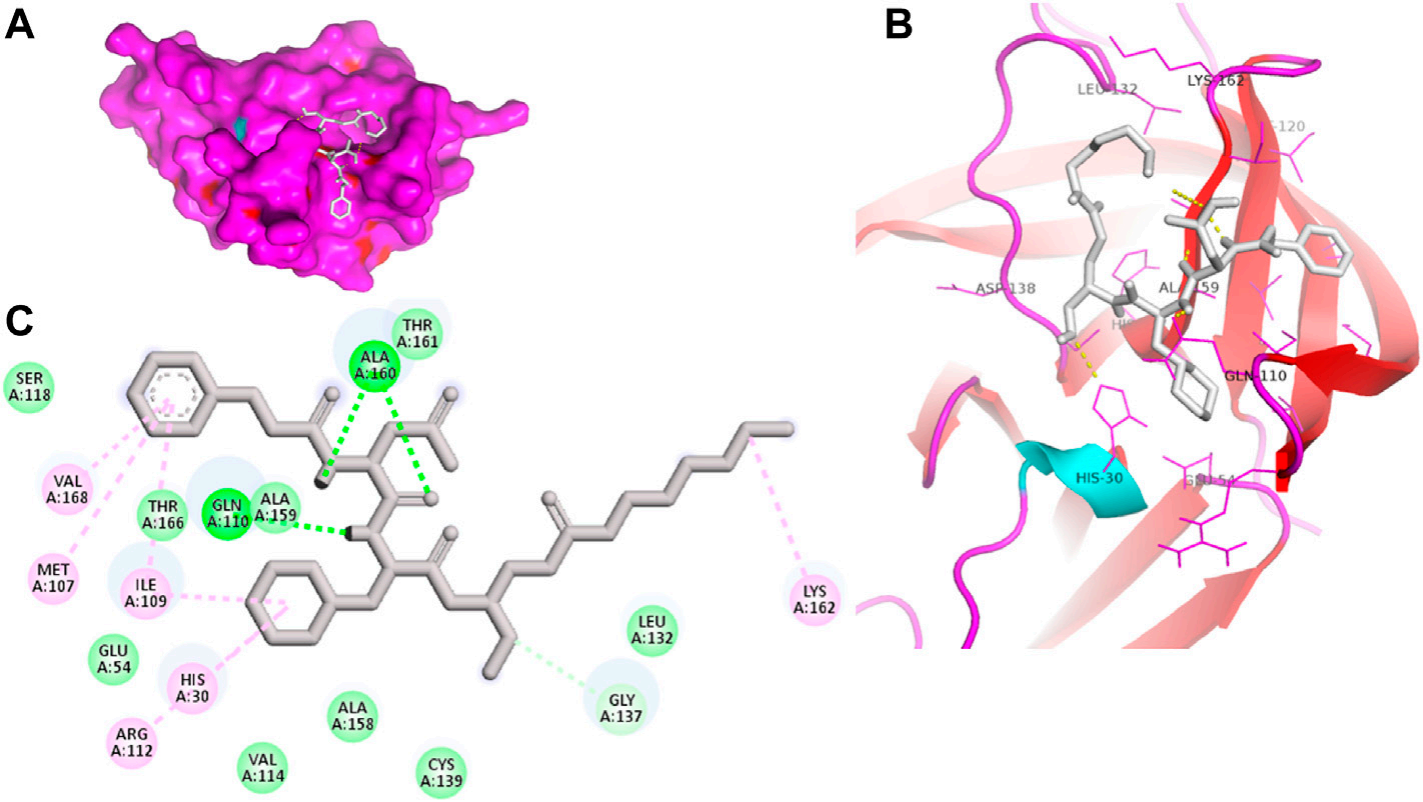
Surface view of dipeptidyl inhibitor seven in the 3CLP binding pocket **(A)**, 3D **(B)** and 2D **(C)** interacting residues of 3CLP with dipeptidyl inhibitor 7.

Moreover, to obtain a better picture of 3CLP interacting residues with leads (sorafenib, LDC4297, and YM201636), 3CLP interacting residues with its co-crystallized inhibitor (dipeptidyl inhibitor seven; PDB ID: 5T6F) were analyzed, which showed that Gln110, Arg112, Val114, His130, Thr134, Glu54, Met107, Arg108, Ile109, Ile135, Pro136, Gly137, Cys139, Lys152, His157, Ala158, Ala159, Ala160, Thr161, Lys162, and Val168 residues were important in binding with dipeptidyl inhibitor 7 (Figure 5). Interestingly, Ile109, Gln110, Ala158, Ala159, Ala160, and Lys162 were the common binding residues of 3CLP with sorafenib, LDC4297, YM201636, and the dipeptidyl inhibitor 7 (Figure 2, 3, 4, and 5), revealing that the binding mode of these compounds in the 3CLP catalytic pocket was similar to that of the reference inhibitor.

The intention of ligand-protein docking is to anticipate the most probable binding modes of the ligand with the catalytic pocket residues of the target protein, with a high BE value (more negative) implying an effective interaction between ‘inhibitor-protein’ complexes (Meng et al., 2011). Sorafenib, LDC4297, and YM201636 have higher BEs than the control dipeptidyl inhibitor 7 (Table 1), indicating that these leads bind to the NV 3CLP strongly.

Despite advances in developing effective antiviral therapies, the currently available antiviral agents have various issues, including high prices, drug resistance, safety, and effectiveness limits (Antonelli and Turriziani, 2012). Further, we predicted the physicochemical, drug-likeness, and ADMET properties of these selected hits. Table 2 shows the bioactivity scores predicted by the Molinspiration web tool for sorafenib, LDC4297, and YM201636. The chemical compound is active if its bioactivity score is greater than 0.0; moderately active if it is between -5.0 and 0.0; and inactive if it is less than -5.0. As per calculated values, the identified hits, Sorafenib, YM201636, and LDC4297 are physiologically active/moderately active substances and meet the criteria mentioned here.

**TABLE 2:** Bioactivity score of top three hits.

Properties		Top 3 hits		
		Sorafenib	YM201636	LDC4297
Ligands	GPCR	0.18	0.03	0.04
	Nuclear receptor	-0.07	-0.81	-0.55
Ion channel modulator	0	-0.38	-0.27	
Inhibitors	Kinase	0.44	0.39	0.36
	Protease	0.11	-0.32	-0.23
	Enzyme	0.08	0.27	-0.11

The pharmacokinetics viability and druglikeness characteristics of identified hits computed using the SwissADME and DataWarrior tools indicated that they should be promising lead candidates. Table 3 displays the values of the different properties, *viz.*, physicochemical properties, pharmacokinetics, and druglikeness of the Sorafenib, YM201636, and LDC4297.

**TABLE 3:** Physicochemical and druglikeness properties of selected hits.

Selected hits		Sorafenib	YM201636	LDC4297	
**Physicochemical Properties**	Molwt	464.83	467.488	432.53	
	cLogP	4.1428	2.7535	1.7767	
	cLogS	-6.689	-7.308	-4.913	
	H-Acceptors	7	10	9	
	H-Donors	3	2	2	
	TPSA	92.35	132.29	94.19	
**Lipophilicity**	iLOGP	3.42	3.08	4.04	
	XLOGP3	4.07	2.46	3.43	
	WLOGP	6.32	2.94	2.45	
	MLOGP	2.91	1.18	2.63	
	Silicos-IT Log P	3.78	2.47	1.65	
	Consensus Log P	4.1	2.43	2.84	
**Water Solubility**	ESOL	Log S	-5.11	-4.49	-4.68
		Solubility (mg/ml)	3.62E-03	1.52E-02	8.97E-03
		Class	Moderately soluble	Moderately soluble	Moderately soluble
	Ali	Log S	-5.71	-4.88	-5.09
		Solubility (mg/ml)	8.98E-04	6.13E-03	3.53E-03
		Class	Moderately soluble	Moderately soluble	Moderately soluble
**Pharmacokinetics**	GI absorption	L	H	H	
	BBB permeant	N	N	N	
	Pgp substrate	N	Y	Y	
	Inhibitor	CYP1A2	Y	Y	Y
		CYP2C19	Y	Y	N
		CYP2C9	Y	Y	Y
		CYP2D6	Y	Y	Y
		CYP3A4	Y	Y	Y
	log Kp (cm/s)	-6.25	-7.41	-6.5	
**Druglikeness**	Violations	Lipinski	0	0	0
		Ghose	1	1	0
		Veber	0	0	0
		Egan	1	1	0
		Muegge	0	0	0
	Bioavailability Score	0.55	0.55	0.55	
	Mutagenic	N	N	N	
	Tumorigenic	N	N	N	
	Reproductive Effective	N	N	N	
	Irritant	N	N	N	
	Druglikeness score	-5.1185	2.2644	4.0968	

(Y=Yes, N=No, None, H=High, L = Low).

Evaluation of pharmacokinetic properties for a success therapeutic in the early stages of drug design by *in silico* ADME assays is critical to achieve druglikeness and decrease risk attrition in the advanced stages. The predicted compounds had acceptable drug-likeness and pharmacokinetic features, and they could not be P-glycoprotein substrates (P-gp). The cytochrome P450 monooxygenase (CYP) enzyme superfamily, which includes cytochrome CYP1A2, CYP2C19, CYP2C9, CYP2D6, and CYP3A4, is crucial in drug metabolism in the liver, and drug biotransformation by O-type oxidation processes, particularly those of 2D6, 2C9, and 3A4, has been anticipated. Sorafenib, YM201636, and LDC4297 were shown to be CYP1A2, CYP2D6, and CYP2C9 inhibitors rather than CYP2C19 inhibitors. These three compounds had substantial lipophilicity and were moderately soluble in water.

**FIGURE 6:**
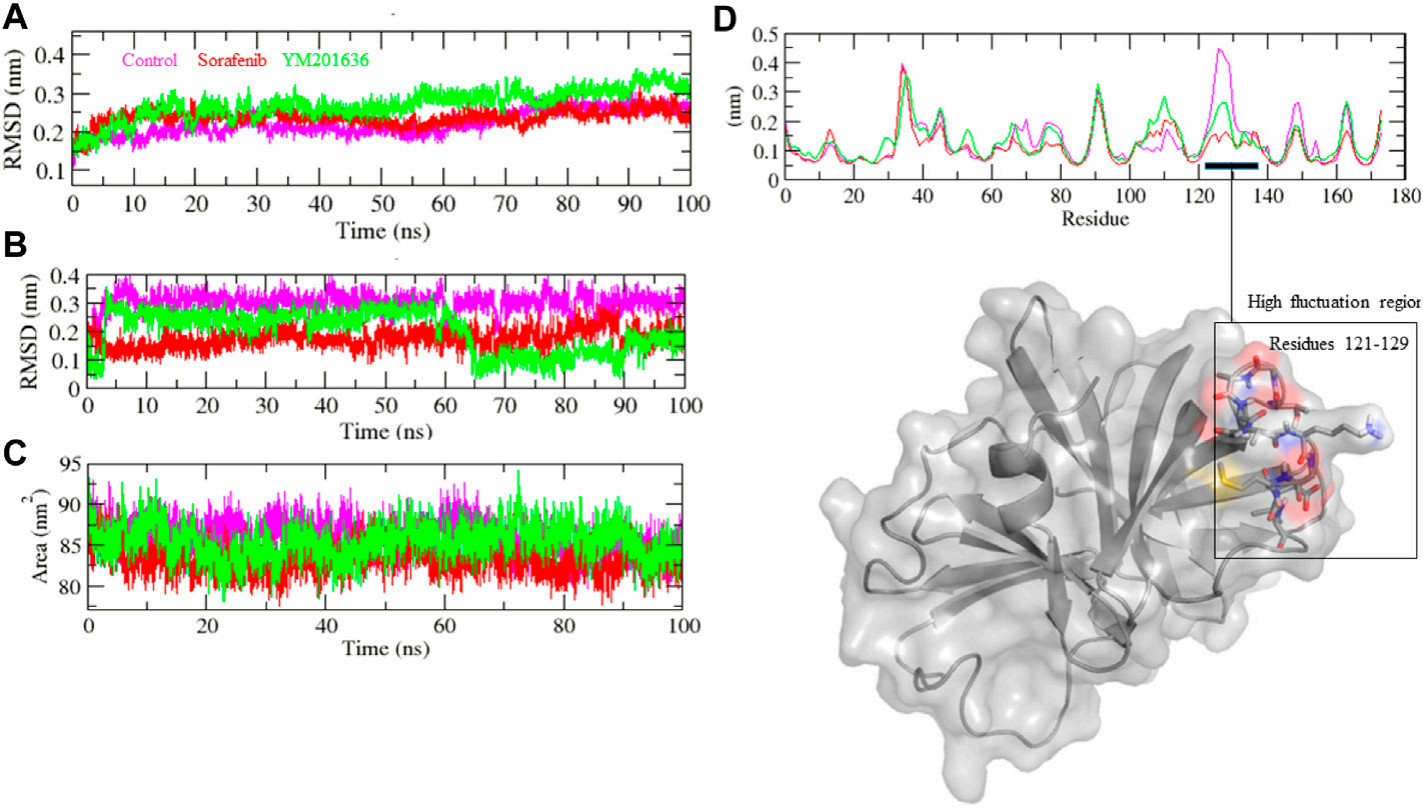
Structural stability studies of complexes. 3CLP-Sorafenib, 3CLP-YM201636, and 3CLP-control complexes were depicted in red, green, and pink color, respectively. RMSD plot of backbone of protein with complexes **(A)**, RMSD plot of ligands within pocket of protein **(B)**, SASA plot of complexes **(C)**, and RMSF plot of backbone of protein **(D)**.

Finally, based on BE, sorafenib and YM201636 including the control were chosen for MD simulation studies to assess the stability of complexes. The root mean square deviation (RMSD) is a protein stability metric; the slighter the deviations, the more stable the protein structure (Aier et al., 2016). 3CLP-control, 3CLP-Sorafenib, and 3CLP-YM201636 had RMSD average values of 0.21, 0.23, and 0.32 nm, respectively. The RMSD figure revealed that 3CLP-control and 3CLP-Sorafenib binding increased 3CLP stability and resulted in fewer structural aberrations from its normal conformation. The bound structure of the 3CLP-YM201636 complex is highly deviated (Figure 6A); it showed that the catalytic pocket of 3CLP not forming well interaction with YM201636, therefore it showed high deviation. In addition, the ligand RMSD also exhibits that control and Sorafenib bind better than YM201636 and they showed more stability (Figure 6A).

The 3CLP-Sorafenib and 3CLP-YM201636 backbones displayed consistent fluctuations in the 3CLP catalytic pocket, most probably due to differing orientations, with the biggest fluctuation regions found between 25–30 and 120–125 residues (Figure 6D). The vibrations around the equilibrium are not random; rather, they are determined by the flexibility of the local structure. The average fluctuation of all residues throughout the simulation, as well as the root mean square fluctuation (RMSF) of 3CLP during binding of 3CLP-control, 3CLP-Sorafenib, and 3CLP-YM201636, were plotted as a function of 3CLP protease residue numbers. The RMSF plot revealed that 3CLP has residual variants in various protein domain areas. Due to their close interaction with the 3CLP. 3CLP-control, and 3CLP-Sorafenib have been shown to reduce the residual fluctuations of the protease.

**FIGURE 7:**
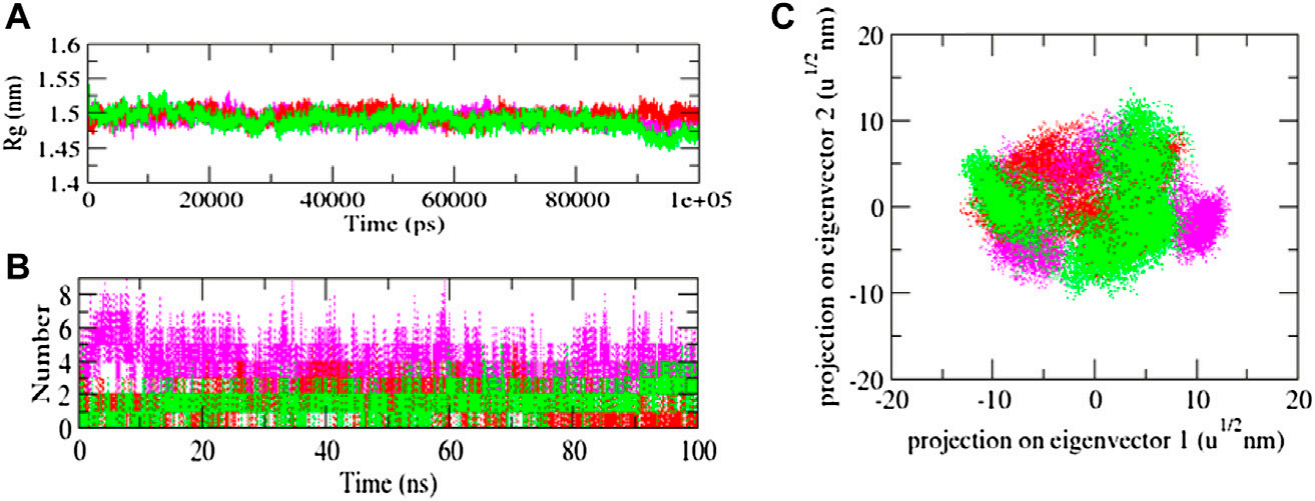
Radius of gyration plot of complexes **(A)**, Hydrogen bond interaction **(B)**, and 2D plot of projection **(C)**.

The solvent-accessible surface area (SASA) of a protein is the area of its surface that is involved in the interaction with its solvent molecules. Average SASA values for 3CLP-control, 3CLP-Sorafenib, and 3CLP-YM201636 complexes were recorded during the 100 ns simulation. The SASA values for the 3CLP-control, 3CLP-Sorafenib, and 3CLP-YM201636 complexes were 88.02, 84.01, and 85.30 nm^2^, respectively (Figure 6C). SASA analysis showed that upon binding of Sorafenib, surface exposure has been reduced. Further to gain insight of the complex stability/compactness profile in a biological system, we applied the Radius of gyration (Rg). The 3CLP-control, 3CLP-Sorafenib, and 3CLP-YM201636 complexes had average Rg values 1.50, 1.53, and 1.47 nm, respectively. Stable Rg trajectories were observed within the catalytic pocket of 3 C L protease (Figure 7A).

The hydrogen bond is vital to the stability of the ligand-target complex (Hubbard and Kamran Haider, 2001). Between protein and ligand, hydrogen bonds were formed within 0.35 nm. The stability of docked complexes was tested using 100 ns simulations of 3CLP-control, 3CLP-Sorafenib, and 3CLP-YM201636 in a solvent environment. 3CLP-control, 3CLP-Sorafenib form 3-6 hydrogen bonds with the 3CLP catalytic pocket, whereas 3CLP-YM201636 forms 1-2 hydrogen bonds with the 3CLP catalytic pocket (Figure 7B).

**FIGURE 8:**
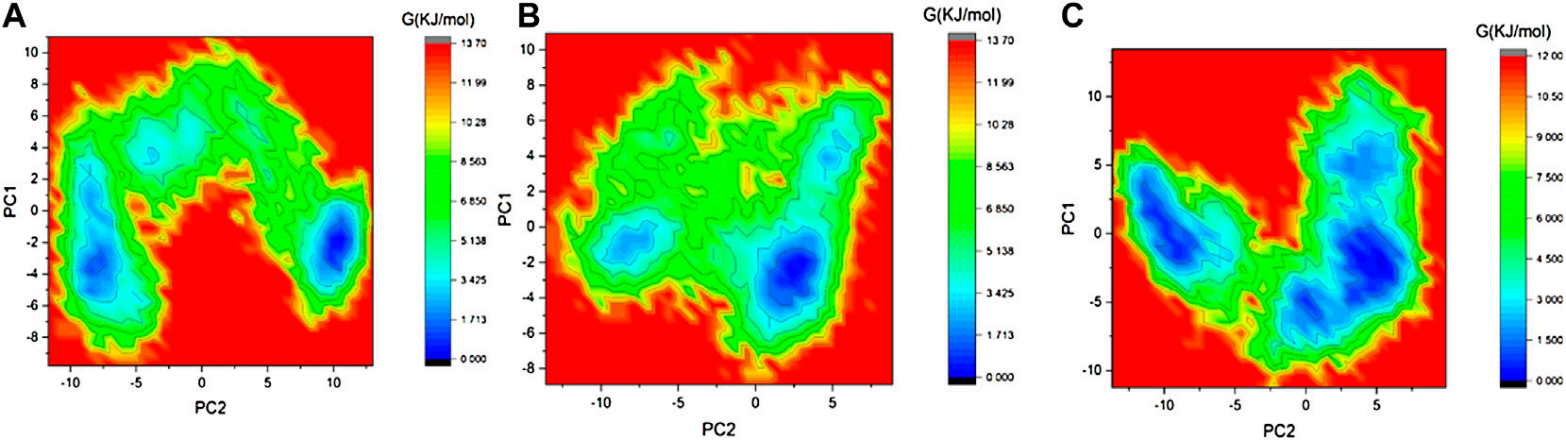
GFE landscape of 3CLP-control **(A)**, 3CLP-Sorafenib **(B)**, and 3CLP-YM201636 **(C)** complexes.

The Gibbs free energy (GFE) landscape of the 3CLP -control, 3CLP-Sorafenib, and 3CLP-YM201636 complexes has been plotted. The blue color represents the location with the least amount of energy. The 3CLP-control and 3CLP-Sorafenib complexes have two distinct global energy minima basins (in blue) (Figures 8A,B), but the 3CLP-YM201636 complexes have three global energy minima states (Figure 8C). More blue spots imply changes in the protein structure followed by a thermodynamically more favorable state, whereas increased blue areas suggest more stability.

## Conclusion

Given the clinical relevance of NV, the study presented here focuses on a high-throughput virtual screen of an antiviral natural compound library against the NV 3CLP using computational technique. The lead compounds sorafenib, LDC4297, and YM201636 met ADMET criteria and interacted with key 3CLP residues. Based on BE, sorafenib and YM201636 were chosen for MD simulation studies, and these compounds displayed stability with the 3CLP. Therefore, these compounds have the potential to be useful in the development of 3CLP inhibitors, and further testing in wet laboratory is warranted.

## Data Availability

The original contributions presented in the study are included in the article/Supplementary Material, further inquiries can be directed to the corresponding authors.
