# Supplementary material for: Identification of compelling inhibitors of human norovirus 3CL protease to combat gastroenteritis: A structure-based virtual screening and molecular dynamics study
Source: Front Chem. 2022 Sep 30;10:1034911. doi: 10.3389/fchem.2022.1034911 (PMC9561104; doi:10.3389/fchem.2022.1034911)
Supplement: Supplementary file 1 [file Table1.docx]

Identification of compelling inhibitors of human norovirus 3CL protease to combat gastroenteritis: A structure-based virtual screening and molecular dynamics study

Shan He^1,2,3^, Alaa F. Nahhas^4^, Alaa Hamed Habib^5^, Mohammed Ali Alshehri^6^, Saleh Alshamrani^6^, Saeed A. Asiri^6^, Mashael M. Alnamshan^7^, Nawal Helmi^8,9^, Ibtesam Al-Dhuayan^10^, Jawaher Almulhim^11^, Ahmed M. Alharbi^12^, Dongxiao Su^1*^, Ankita Kumari^13*^and Abdul Rahaman^13*^

| Ligand | Affinity (kcal/mol) |
| --- | --- |
| Sorafenib | **-11.67** |
| YM201636 | **-10.34** |
| LDC4297 | **-9.78** |
| DBeQ | -8.71 |
| KPT-335 | -8.43 |
| CB-5083 | -8.40 |
| Raltegravir | -8.30 |
| OSU_03012 | -8.10 |
| Salubrinal | -7.65 |
| LY2784544 | -7.51 |
| GNF-2 | -7.44 |
| GSK650394 | -7.36 |
| PD-0332991_hydrochloride | -7.33 |
| Camptothecine | -7.25 |
| Cinacalcet_Hydrochloride | -7.25 |
| FR_180204 | -7.22 |
| Purvalanol_A | -7.12 |
| FM-381 | -6.54 |
| Dipeptidyl inhibitor 7 | -6.38 |

**Table S1.** Top 20 screened compounds including control (Dipeptidyl inhibitor 7) and their BE with 3CLP.
